# Thrombosis in Chronic Kidney Disease in Children

**DOI:** 10.3390/diagnostics12122931

**Published:** 2022-11-24

**Authors:** Tudor Ilie Lazaruc, Lavinia Bodescu Amancei Ionescu, Vasile Valeriu Lupu, Carmen Muntean (Duicu), Roxana Alexandra Bogos, Anca Ivanov, Georgiana Scurtu, Iuliana Magdalena Starcea, Ingrith Crenguta Miron, Maria Adriana Mocanu

**Affiliations:** 1 Department of Pediatrics, “Grigore T. Popa” University of Medicine and Pharmacy, 16 Universitatii Street, 700115 Iasi, Romania; 2 Department of Pediatrics I, George Emil Palade University of Medicine, Pharmacy, Science and Technology of Targu Mures, 540088 Targu Mures, Romania; 3 Nephrology Division, St. Mary’s Emergency Children Hospital, 700309 Iasi, Romania

**Keywords:** venous thromboembolism, chronic kidney disease, child

## Abstract

Venous thromboembolism (VTE) in children is a rare condition. An increased incidence has been observed in the last few years due to several factors, such as increased survival in chronic conditions, especially chronic kidney disease (CKD), use of catheters, and increased sensitivity of diagnostic tools. VTE includes deep vein thrombosis (DVT) and pulmonary embolism (PE). VTE in children is associated with a two to six times higher mortality risk and a 5–10% prevalence of post-thrombotic syndrome. Overall, 5% of VTE episodes in children are associated with chronic kidney disease. The etiology of VTE in chronic kidney disease covers a wide range of pathologies. Various types of thrombotic complications may occur during long-term use of a chronic dialysis catheter. VTE occurs in 3% of children with nephrotic syndrome (NS). The risks for VTE and arterial thromboembolism (ATE) were particularly high in the first 6 months after the onset of NS. Other causes of VTE are graft rejection due to thrombosis of vascular anastomoses after kidney transplantation (3%) and autoimmune diseases (lupus nephritis, antiphospholipid syndrome). In this state-of-the-art overview, we have reviewed the physiologic and pathologic mechanisms underlying pediatric thrombosis and updated current diagnostic and treatment options, emphasizing personal experience as well.

## 1. Introduction

Chronic kidney disease (CKD) is a pathological entity burdened by numerous complications with a wide spectrum of clinical and paraclinical manifestations. Etiologically, CKD is caused by glomerular or non-glomerular diseases, and in the pediatric population, it is frequently associated with congenital kidney and urinary tract malformations (Congenital Anomalies of Kidneys and Urinary Tracts—CAKUT) or hereditary nephropathies. Among the complications associated with this complex pathology, thromboembolism (VTE) is an important source of mortality and morbidity, with multiple etiopathogenic mechanisms involved in its occurrence. The prompt diagnosis and treatment of VTE requires a high index of suspicion corroborated with specific blood analyses and imaging investigations. The children with CKD and suspected VTE require special diagnostic strategies, such as anesthesia, scheduling the hemodialysis session, adjusting the doses of radiotracer, anticoagulants, or other specific therapies. In adult patients with suspected VTE, several prediction scores (Wells PE, Wells DVT, revised Geneva score, PERC-score) were designed. The scores offers a good sensitivity and specificity but cannot rule out the VTE diagnosis; instead, they are important to determine the high or low probability of VTE [1,2].

For children with suspected VTE, there are no validated scores or other type of tool. Several studies extrapolated these scores, Wells and PERC, to the pediatric patients, but no predictive score were validated [3,4]. Children with CKD present an increased risk of VTE, and special attention must be directed to possible recurrences.

The VTE diagnosis in adult patients benefits from multiple tools, but the occurrence of recurrences requires detailed and relevant imaging methods [5]. Children, especially those with dialysis, require special management before and after imaging investigations such as CT angiography. Thrombelastogram and thrombin generation test are global hemostasis tests that provide information on both bleeding and clotting tendencies. Thrombelastogram has been used in the management of extracorporeal circuits in ESRD. Measurement of antithrombin (AT), protein C, and protein S levels, as well as the identification of factor V Leiden or prothrombin gene mutation, only provide information about a specific component of the hemostatic system, but they do not allow quantification of the general thrombotic tendency resulting from the interaction between inherited and acquired. The AT level can be influenced by heparin therapy, while the activity of protein C and protein S can be falsely decreased during vitamin K antagonist therapy [6]. Low levels are also found during the state of the nephrotic syndrome, explaining the tendency to hypercoagulability of these patients. APTT is used to evaluate the effect of therapy with unfractionated heparin. To assess the anticoagulant effect of fractionated heparin or some of the newer direct-acting oral anticoagulants (DOACs), the anti-Xa assay or activated clotting time (ACT) is routinely used to assess bleeding/thrombosis risks (2). The level of D-dimers is a measure of fibrin production and degradation. It has been used in adults in pulmonary embolism diagnostic algorithms. Normal pediatric ranges are age dependent [6]. However, global coagulation tests do not assess the significant role played by platelets and endothelial cells. Platelet function can be assessed by identifying markers of platelet activation and reactivity in plasma and/or on circulating platelets, such as platelet factor 4 and β-thromboglobulin in platelet-poor plasma. This is usually performed by blood flow cytometry, which is used to measure activated platelets, as well as platelet turnover [7]. Consequently, newer assays that directly measure thrombin generation in plasma, as well as those that assess the stages of hemostasis, including clot initiation, propagation, and fibrinolysis in whole blood by viscoelastic methods, are what may allow a global measurement of the hemostatic system. Currently available methods for viscoelastic testing include thromboelastography (TEG) and the related rapid thromboelastography (r-TEG) as well as rotational thromboelastography (ROTEM) [7].

The treatment with the new direct oral anticoagulants (DOACs) in children are not yet considered a standard of care, especially in children with renal impairment because of increased risk of bleeding complications. Several studies in the field, such as the DIVERSITY and EINSTEIN-Jr studies, ruled out the children with various degrees of renal failure. DOACs have many advantages and, in general, the patients require a normal renal function. Although the renal excretion of every type of DOACs is different, the administration in patients with CKD is limited [8,9].

## 2. Epidemiology

While in the general pediatric population, the incidence of VTE is only 0.07–0.49 cases/10,000 children, in children hospitalized for any cause, it reaches 4.9–21.9 cases/10,000 children [10]. This reveals the importance of VTE in the course and prognosis of patients during hospitalization and indicates that in the pediatric patient, the susceptibility for the development of VTE is closely related to the presence and progression of underlying pathologies.

The mortality risk of deep vein thrombosis (DVT) and pulmonary thromboembolism (PTE) as constituent entities of VTE is 2–6 times higher than in the general pediatric population [10], and these patients also associate a 5–10% prevalence of post-thrombotic syndrome (disabling venous insufficiency) with the development of recurrent VTE in 10% of cases [10].

Literature data also show that the incidence of VTE has a bimodal distribution: up to 20% of cases constitute a first peak occurring in the neonatal period, and up to 50% of cases are responsible for a second peak occurring during adolescence and puberty [8]. Part of the explanation for this distribution lies in the physiologically low level of anticoagulant factors in the neonatal period and reduced fibrinolytic activity at puberty [11].

**Figure 1:**
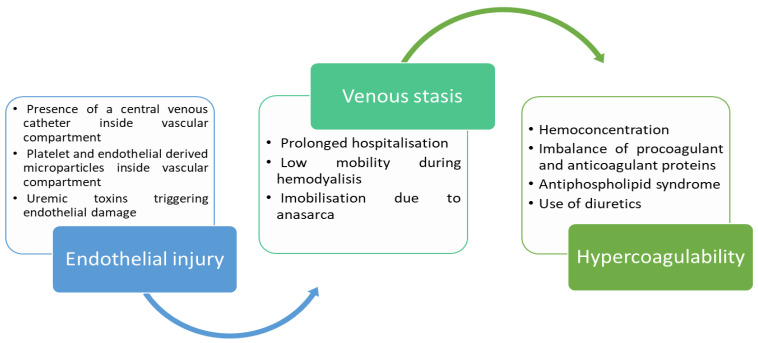
Renal involvement in Virchow’s triad.

However, it is necessary to take into account that the increase in the incidence of VTE at this age is linked to the progression of chronic renal pathologies. Biological manifestations lead to the emergence of the specific pathophysiological context of VTE occurrence, which is conceptualized in the form of Virchow’s triad (Figure 1). CKD favors the onset of this triad (consisting of venous stasis, endothelial injury and hypercoagulable status) by various mechanisms, which is why, compared to other pediatric chronic conditions, CKD has the highest rate of hospital-associated VTE [12]. However, this association differs according to the etiology of the CKD. An analysis of 22,877 patients with CKD conducted between April 2003 and June 2012 revealed an overall VTE rate of 0.55%, of which 0.32% is in patients with reno-urinary malformations, 1.93% is in patients with nephrotic syndrome, 2.74% is in transplanted End-Stage Renal Disease (ESRD) patients, and 6.32% is in hemodialysis patients [13]. The graft failure rate due to thrombosis of vascular anastomoses following renal transplantation is approximately 3% [14] and the estimated thrombus-free survival of arteriovenous fistulae (AVF) for long-term hemodialysis is 74% at 1–2 years [15]. However, the distribution and determinants of VTE (including VTE secondary to the graft failure, and VTE in AVF) have not yet been established for children with CKD.

Thus, VTE is a severe complication in CKD of various etiologies, which is driven by complex and different mechanisms involving numerous risk factors. The main studies investigating this pathology in children are systematized below in Table 1.

## 3. VTE and Nephrotic Syndrome

Nephrotic syndrome (NS) is associated with a hypercoagulable state caused by thrombocytosis and numerous hemostatic abnormalities: on the one hand, low levels of antithrombin III, free protein S and plasminogen in the context of urinary leakage, and on the other hand, increased levels of procoagulant proteins (fibrinogen and factors V and VIII) with consequent increased platelet activation. Pathophysiologic factors involved in the increased risk of thromboembolic complications in children with NS also include hemoconcentration (Hb greater than 14 g/dl), dehydration, immobilization (especially in patients with anasarca), infection, venous or arterial puncture (CVC), diuretic consumption, corticosteroids (i.v.), thrombocytosis (over 450,000/mm^3^), proteinuria, hypoalbuminemia (below 20 g/dl), hyperfibrinogenemia as well as a possible underlying genetic thrombophilic tendency.

Although the reported incidence of thromboembolic complications in children with NS is between 1.8 and 4.4% [16], in patients with congenital NS, the incidence of VTE can be as high as 10–13% [17]. This is consistent with the results of a retrospective study including 326 children with NS of different etiologies, conducted between 1999 and 2006, in which the incidence of thromboembolic events (TEE) was reported to be 9%, with a mean time to TEE of approximately 71 days after diagnosis of NS and with DVT as the most common entity seen in these children, which is closely correlated with CVC use [18].

The underestimation of the true incidence of thromboembolic complications in children with NS is, however, supported by other evidence. Investigation by ventilation-perfusion scintigraphy of 26 children with NS in remission or with minimal symptoms identified evidence of new-onset PTE in 7 (27%) of these, with a further 10 (38%) showing evidence of previous PTE [19]. Multivariate analysis demonstrated that the risk of TEE was higher in children over 12 years of age and increased with increasing urinary protein excretion.

Another recent study of 512 patients, 80 of whom were children, with NS either in remission or with minimal symptoms detected PE ± recurrent venous thrombosis (RvT) in 35% of cases (predominantly PE—85%), 19% of the pediatric group associated PE ± RvT [18]. All these aspects suggest that subclinical VTE associated with NS may be much more common than commonly appreciated.

Regarding the etiology of NS in relation to VTE, a retrospective study on a group of children with secondary NS (including autoimmune glomerulopathies) found that they were associated with a higher risk of developing VTE versus those with primary NS (17.0 vs. 6.6%) [18]. The category of secondary NS includes systemic lupus erythematosus (SLE) and IgA vasculitis, which associate antiphospholipid antibody production as well as a generalized inflammatory status that may biologically mediate the increased risk of VTE in these patients.

Regarding the location of thromboembolism in patients with NS, both arterial and venous thrombosis have been reported, with the latter predominating and occurring most frequently in the cerebral and pulmonary veins. The cerebral symptoms may lend themselves to a differential diagnosis with posterior reversible encephalopathy syndrome (PRES), especially in the case of patients treated with immunosuppressive drugs such as calcineurin inhibitors. We have previously presented a case of PRES in a patient with Focal segmental glomerulosclerosis (FSGS), manifested by nephrotic syndrome, who developed neurological symptoms and in which neuroimaging established the final diagnosis of PRES. The girl associated nephrotic-range proteinuria and hypercoagulability with elevated D dimer values [20].

The other locations reported were pulmonary artery, renal vein, deep leg veins, superior vena cava and inferior vena cava, femoral arteries and iliac arteries [18].

**Figure 2:**
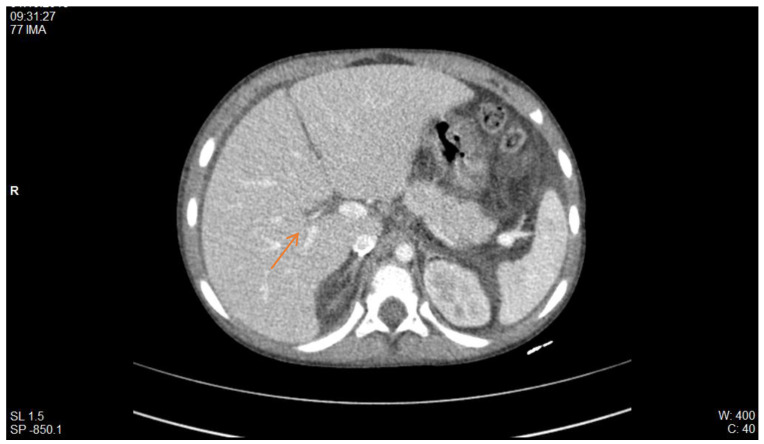
CT scan: thrombosis of the inferior vena cava with extension to the right renal vein (red arrow) (personal collection Nephrology Department).

In the Nephrology Division, St. Mary’s Emergency Children Hospital, we have been confronted with thrombotic pathology in patients with nephrotic syndrome. Of particular relevance is the case of a male patient, diagnosed with cortico-resistant nephrotic syndrome, in whom renal biopsy detected minimal glomerular lesions and IgM deposits. In evolution, he presented multiple relapses marked by massive oedema, requiring correction with albumin and diuretic therapy. In this context, he developed thrombosis of the inferior vena cava, as shown by CT imaging, with extension to the right renal vein and to the junction of the internal and external iliac veins (Figure 2). In evolution, he developed three more episodes of thrombotic recurrence, under the same conditions of severe hypoproteinemia, and required 2 years of anticoagulant therapy.

**Figure 3:**
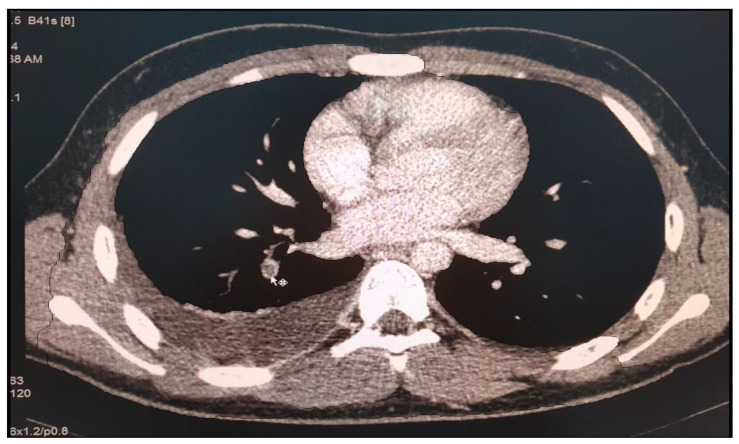
CT scan, appearance of right pulmonary artery segmental branch thrombosis, pulmonary embolism and right secondary pleurisy (personal collection Nephrology Department).

Another case is of a female patient diagnosed with SLE and class IV lupus nephritis, who was initially treated with pulse therapy with methylprednisolone and i.v. cyclophosphamide. After 4 months of evolution, she presented with fever and acute respiratory failure phenomena for which we was excluded an infectious pathology (tuberculosis, viral, fungal or bacterial pneumonia). Biologically, the antiphospholipid syndrome was detected with high titer anticardiolipin antibodies (73.96 pg/mL), and the presence of lupus anticoagulant. CT imaging examination (Figure 3) revealed the appearance of right pulmonary artery segmental branch thrombosis, pulmonary embolism and right secondary pleurisy. Anticoagulant treatment with Enoxyparin was instituted for 6 months, at the end of which respiratory function tests revealed severe restriction and medium obstruction (CV = 47.6%, FEV 1 = 54.8%). Oral anticoagulant therapy was maintained under INR control for 2 years.

VTE is associated with significant morbidity and mortality in children with NS, with an incidence approaching 25% in high-risk groups. The etiology of VTE associated with this pathology is multifactorial, the associated coagulopathy having a significant contribution. Other risk factors include age and severity of the disease. Further studies are needed to identify VTE risk biomarkers and optimal therapeutic regimens in patients with chronic nephropathy.

## 4. VTE and ESRD

ESRD corresponds to an estimated filtration rate (eRFG) defined by creatinine clearance of less than 15 mL/min/1.73 m^3^ over a minimum of 3 months. Children with ESRD frequently show imbalances in hemostasis with the risk of consecutive bleeding or pathological thrombosis. Factors that increase the risk of VTE in ESRD are correlated, on the one hand, with the underlying pathology that caused the progression of CKD and, on the other hand, with ESRD-specific uremia. In this regard, uremic toxins derived from tryptophan catabolism stimulate thrombotic processes mainly by increasing platelet activation (in the context of increased fibrinogen receptor levels and decreased nitric oxide levels) and by the constitution of a “prothrombotic” endothelium (predominantly in the context of homocysteine-mediated endothelial cell injury, the formation of an intravascular reservoir of platelet- and endothelium-derived microparticles and the consequent increase in tissue factor concentration).

Regarding the renal replacement methods to be used in these patients, although peritoneal dialysis is preferred in young children, hemodialysis is the most common method in the 0–21 age group. Vascular access for this can be via arteriovenous fistula (AVF), subcutaneous tunneled CVC or synthetic arteriovenous graft implantation. In the context of vascular fragility often encountered in the pediatric patient, the most common access route involves the use of a CVC. However, its presence in the vascular lumen is thrombogenic, but this effect is influenced by the material from which the CVC is made (polyurethane more than silicone), the type of catheter (peripherally inserted CVC more than non-tunneled CVC) and the technique used for its insertion (classical anatomical approach more than ultrasound-guided approach).

**Figure 4:**
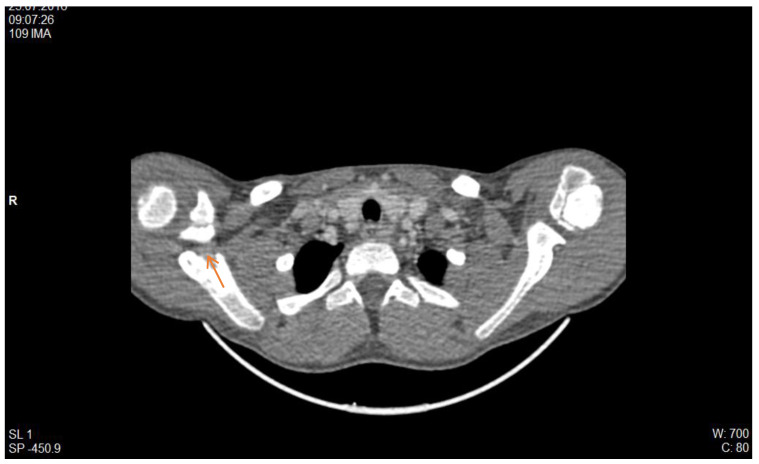
CT scan, right IJV thrombosis (red arrow) (personal collection Nephrology Department).

From the experience of the Nephrology Division, St. Mary’s Emergency Children Hospital, Iasi, we illustrate with the case of a male patient diagnosed with recurrent urinary tract infections due to a late detected posterior urethral valve, which is associated with secondary vesicoureteral reflux. He required initiation of dialysis on long-life central catheter in January 2016. In evolution, he presented two episodes of catheter dysfunction through thrombosis, for which we initiated local therapy with Taurolok/Urokinase. Subsequently, he required catheter replacement because of septic complications. A new thrombotic episode was observed in the evolution, which was biologically supported by increased serum D-dimer level (2314 ng/mL) and confirmed by computer tomographic (CT) examination (Figure 4), with localization in the right internal jugular vein (IJV).

**Figure 5:**
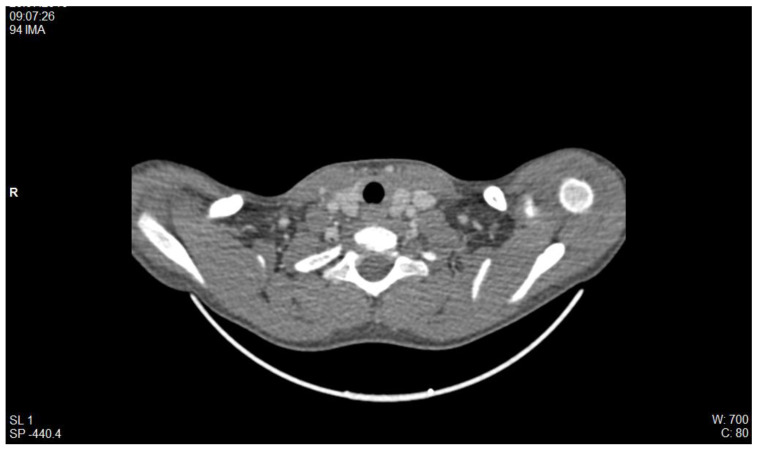
CT scan, evaluation 2 months after the acute episode reveals persistence of thrombosis in the right IJV without progression (personal collection Nephrology Department).

In this context, thrombolytic treatment with tissue plasminogen activator factor was initiated, and further hemodialysis sessions required the placement of a temporary CVC on the right femoral vein. To prevent further thrombosis, anticoagulation was continued with enoxaparin in a dose adapted to the creatinine clearance. After 2 months, the patient was evaluated by angio-CT (Figure 5), noting the persistence of venous thrombosis in the right IJV. At that time, it was decided to place a long-life CVC on the left IJV, on which hemodialysis is still performed.

**Figure 6:**
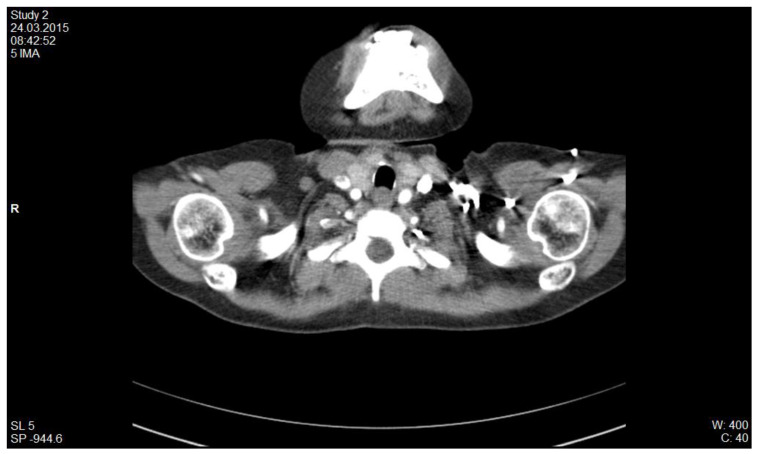
CT scan, right IJV thrombosis, calcifications in the trachea in the context of calciphylaxis.

Another case in our clinic’s collection was a female patient diagnosed with impure nephrotic syndrome with unfavorable progression to end-stage chronic kidney disease. She underwent renal replacement therapy by continuous ambulatory peritoneal dialysis for 13 years but required conversion to chronic hemodialysis in the context of sclerosing peritonitis. One year after conversion, she presented with an episode of catheter dysfunction, with CT scanning revealing right IJV thrombosis (Figure 6) as well as tracheal calcifications in the context of calciphylaxis. Subsequently, hemodialysis proceeded with difficulty, with the patient evolving numerous thrombotic recurrences on the newly placed catheter.

**Figure 7:**
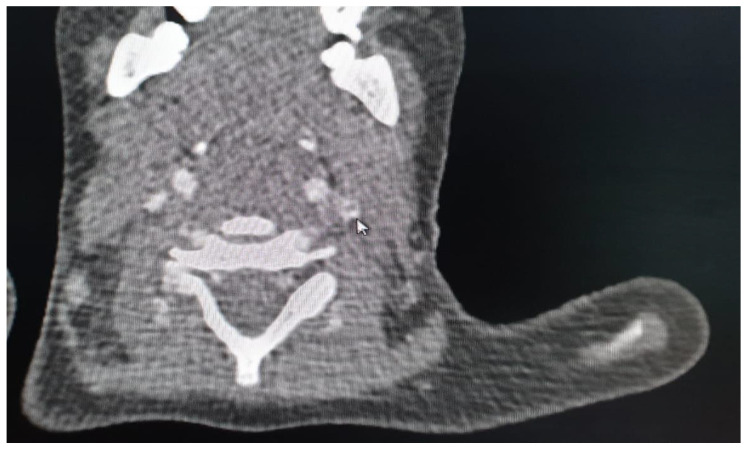
CT scan, left IJV thrombosis. (white arrow).

**Figure 8:**
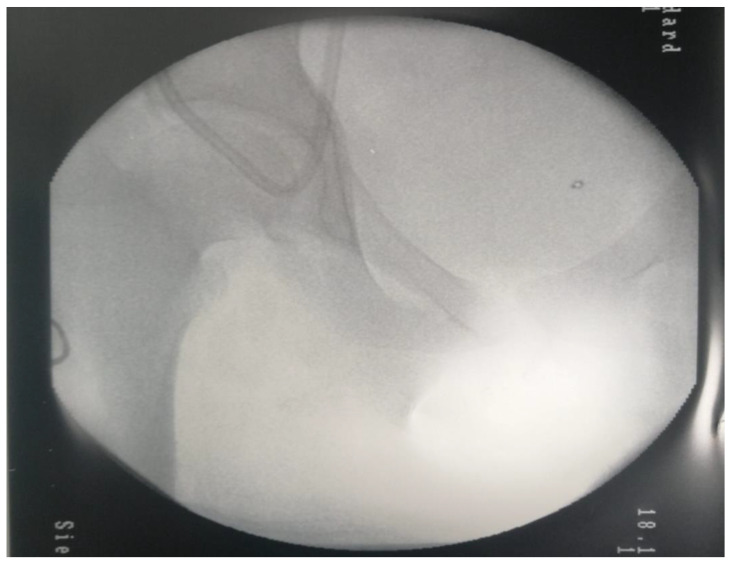
C arm X-ray—Unusual placement of CVC in the epigastric vein for chronic hemodialysis.

A third case presented is a girl who has reached the end stage of kidney disease due to a reflux nephropathy. In evolution, she presented multiple episodes of peritonitis, with secondary sclerosing of peritoneus, necessitating conversion to hemodialysis on CVC. In the context of a heterozygous profile for thrombophilia, the girl developed numerous episodes of CVC thrombosis, which is a dysfunction that prejudiced the dialysis sessions. In the context in which she developed extensive thrombosis of the bilateral jugular vein and brachycephalic trunk (Figure 7), the girl required an unusual vascular approach, with access to the iliac vein, through the Seldinger technique on the epigastric vein (Figure 8). The literature confirms that factor V Leiden and prothrombin G20210A are considered to be predominant genetic risk factors for VTE in Caucasian populations, and heterozygotes have a 20-fold increased risk of VTE, while individuals with the prothrombin G20210A allele had an increased about four times risk of thrombosis [21].

Chronic kidney disease patients are at high risk of venous thrombosis, and the underlying mechanisms are still unclear and may be attributed to various etiologies such as abnormal platelet activation and accumulation, activation of the coagulation system, decreased endogenous anticoagulants, and decreased activity of the fibrinolytic system.

## 5. VTE and Kidney Transplantation

For pediatric ESRD patients undergoing renal transplantation, a common cause of graft rejection is renal artery or vein thrombosis. According to the 1995 North American Pediatric Renal Trials and Collaborative Studies (NAPRTCS) annual report [22], vascular thrombosis accounts for 12.2% of causes of graft loss, and in the case of second transplantation, vascular thrombosis is blamed for 19.2% of graft loss, replacing acute rejection as the second most common cause of graft failure. Subsequently, the same working group conducted a study of 4394 transplanted children, 138 of whom experienced vascular thrombosis resulting in graft loss [23]. Vascular thrombosis is usually seen within the first few days to three weeks after transplantation. In this specific setting, VTE may occur secondary to technical complications such as torsion or vascular endothelial injury due to excessive manipulation. Risk factors for VTE in this category of patients are age under 2 years of the recipient, age under 6 years of the donor, long cold ischemia time (>24 h), hypoperfusion in young children receiving an adult graft, venous malformation in the recipient, pre-transplant peritoneal dialysis, a hypotensive episode during or after surgery, the presence of multiple arteries as well as previous cyclosporine therapy.

Management of the listed factors involves careful hemodynamic monitoring of central venous pressure to ensure adequate allograft perfusion, correction of vascular depletion related to nephrotic syndrome, screening for inherited and acquired thrombophilia risk factors and specific prophylactic therapy. Adjacent to the use of low molecular weight heparins, a retrospective study of the NAPRTCS database of transplant patients between 1998 and 2004 detected a decreased risk of graft thrombosis in the context of administration of interleukin-2 receptor antagonists [24].

## 6. Conclusions

VTE is a serious complication of CKD and requires increased attention both for the management of acute episodes and for prophylactic measures when necessary. Of the CKD etiologies, NS is associated with the highest risk of thromboembolic events, and risk factors for VTE in children with NS are age over 12 years, history of VTE prior to diagnosis of NS, secondary nephrotic NS (SLE, IgA vasculitis) but also membranous nephropathy. Further research is needed to assess the impact of childhood NS and on vascular endothelial cell biology, which is suspected to play a major role in VTE progression. Observational cohort studies are needed to validate VTE risk groups that may benefit most from thromboprophylaxis and disease-specific treatment algorithms for SN-associated VTE await evidence from multicenter collaborative group studies.

Pediatric ESRD patients represent 1–2% of the general ESRD population. In them, uremia has a thrombogenic effect that overlaps with risk factors for VTE associated with the underlying pathology. In addition, performing hemodialysis on CVC is the most commonly used method of renal supplementation in these patients, and the presence of CVC in the vascular lumen increases the risk of TEE, with CVC occlusion or thrombosis being the main cause of CVC dysfunction.

In kidney transplant patients, vascular thrombosis is the third leading cause of rejection and is most commonly seen in the first few days after transplantation. Despite all the issues described, extensive and validated studies are needed in children with CKD to quantify and identify thromboembolic risk factors. Subsequently, susceptible patients should benefit from non-pharmacological TEE prevention measures or should be enrolled in clinical trials to verify the impact of initiating thrombo-prophylactic therapies.

## Figures and Tables

**Table 1:** Relevant studies depicting the children risk for VTE.

Autors	Study Type	Sample Size and Age	Aims	Main Results
Hennelly KE, Baskin MN, Monuteuax MC, et al., 2016	Single-center retrospective study	561 children < 22 years of age	To evaluate the children risk for PE, using adult Wells criteria and Pulmonary Embolism Rule-out Criteria (PERC)	The risk of pulmonary VTE is low among children not receiving estrogen therapy and without tachycardia and hypoxia. Application of the PERC rule and Wells criteria should be used cautiously in the pediatric population.
Biss TT, Brandão LR, Kahr WH et al., 2009	retrospective cohort study	50 children with PE	To evaluated D-dimer value and Wells probability score for PE in children	The Wells clinical probability score and D-dimer estimation may lack utility in the determination of pre-test probability of PE in children.
Van Ommen CH, Heijboer H, Büller HR et al., 2001	prospective 2-year registry of VTE in children	99 children ≤ 18 years old	To study the incidence, diagnostic, and complications of pediatric VTE	VTE is mostly diagnosed in hospitalized children, especially sick newborns with central venous catheters and older children with a combination of risk factors.
Setty BA, O’Brien SH, Kerlin BA., 2012	The Kids’ Inpatient Database 2006	4500 children ≤ 18 years old	To evaluated the incidence of VTE in tertiary care settings	Pediatric VTE is most commonly seen in tertiary care. Adolescents are at greatest risk to develop in-hospital VTE.
Suri D, Ahluwalia J, Saxena AK et al., 2013	retrospective study	34 children	To evaluated the incidence of venous and arterial thrombosis in children with nephrotic syndrome	Venous and arterial thrombosis occur in children with nephrotic syndrome, with subtle clinical features. Neuroimaging and angiographic techniques confirm diagnosis, and early aggressive heparin therapy is necessary for a favorable outcome.
Zhang LJ, Zhang Z, Li SJ et al., 2014	prospective study	512 patients in the study cohort, 80 children	To determine the prevalence PE and renal vein thrombosis in patients with NS	PE pulmonary embolism and RVT renal vein thrombosis are common in patients with NS, occurring in 19% of children and 38% of adults. PE pulmonary embolism is more common than RVT renal vein thrombosis
Kerlin BA, Blatt NB, Fuh B et al., 2009	comprehensive chart review	326 children	To identify the risk factors of VTE in children with NS	Children with NS have risk for VTE, particularly those who are age 12 years or older, have severe proteinuria, or have a previous history of TE.
Hoyer PF, Gonda S, Barthels M et al., 1986	prospective study	16 children	To evaluated the incidence of VTE in children with steroid responsive minimal change nephrotic syndrome	The incidence of thromboembolic complications in children with severe nephrotic syndrome is as high as reported for adults.
Singh A, Stablein D, Tejani A, 1997	The Report of the North American Pediatric Renal Transplant Cooperative Study database	4394 transplanted children	To identify the risk factors for VTE in transplanted children	Living donor transplant with a history of prior transplantation had a significantly higher rate of thrombosis as compared with those who received a primary transplant. Cold ischemia time greater than 24 h in the patient who received cadaver donor kidney increased the risk for thrombosis. The use of antibody induction therapy, donors greater than 5 years of age, and increasing recipient age were factors that decreased the risk for thrombosis.
Smith JM, Stablein D, Singh A et al., 2006	The North American Pediatric Renal Transplant Cooperative Study (NAPRTCS) database	8990 transplanted children	To identify the risk factors for VTE in transplanted children	The use of IL-2 receptor antibodies as induction therapy is associated with a significantly decreased risk of graft failure due to thrombosis.

## Data Availability

All medical records are from Hospital Info Word application of our institution and can be accessed with the manager approval.
